# Enhancing Boron Neutron Capture Therapy (BNCT) with Materials Based on COSAN-Functionalized Nanoparticles

**DOI:** 10.3390/ph18040466

**Published:** 2025-03-26

**Authors:** Albert Ferrer-Ugalde, Amanda Muñoz-Juan, Anna Laromaine, Paula Curotto, Susana Nievas, María Alejandra Dagrosa, Marcos Couto, Rosario Núñez

**Affiliations:** 1 Institut de Ciència de Materials de Barcelona (ICMAB-CSIC), Campus U.A.B., 08193 Bellaterra, Barcelona, Spain; a.ferrer80@hotmail.com (A.F.-U.); amunoz@mbg.au.dk (A.M.-J.); alaromaine@icmab.es (A.L.); 2 Department of Research and Production Reactors, National Atomic Energy Commission (CNEA), Presbitero Juan González y Aragón, 15, Ezeiza B1802AYA, Argentina; curotto@cae.cnea.gov.ar; 3 Department of Boron Neutron Capture Therapy, National Atomic Energy Commission, Buenos Aires C1429BNP, Argentina; susanaisabelnievas@gmail.com; 4 Department of Radiobiology, National Atomic Energy Commission (CNEA), Buenos Aires C1429BNP, Argentina; alejandradagrosa@cnea.gob.ar; 5 Grupo de Química Orgánica Medicinal, Instituto de Química Biológica, Facultad de Ciencias, Universidad de la República, Iguá 4225, Montevideo 11400, Uruguay

**Keywords:** boron clusters, BNCT, metallacarboranes, *C. elegans*

## Abstract

**Background/Objectives**: Boron neutron capture therapy (BNCT) is a promising approach for selectively targeting and destroying malignant cells using ^10^B isotopes. A significant challenge in BNCT lies in the development of efficient boron delivery systems that ensure adequate boron accumulation within tumor cells. This study aims to synthesize, characterize, and evaluate COSAN-functionalized nanoparticles (**NP@I-COSAN**) as a potential boron carrier for BNCT. **Methods**: Hybrid nanoparticles were synthesized by conjugating monoiodinated cobaltabisdicarbollides (**I-COSAN**) to commercially available acrylic polymer-based nanoparticles. Functionalization and cellular uptake were confirmed through FTIR, TGA, UV-Vis spectroscopy, and TEM/EDX analyses. Biocompatibility was evaluated by assessing cytotoxicity in HeLa cells and *C. elegans* as an in vivo model. Intracellular boron uptake was quantified using ICP-MS, with results compared to those obtained with 4-borono-L-phenylalanine conjugated to fructose (**f-BPA**). An in vitro BNCT proof-of-concept assay was also performed to evaluate therapeutic efficacy. **Results**: **NP@I-COSAN** demonstrated low cytotoxicity and efficient internalization in cells. ICP-MS analysis revealed stable boron retention, comparable to traditional boron agents. The BNCT assay further showed that **NP@I-COSAN** was effective in inducing tumor cell apoptosis, even at lower boron concentrations than conventional treatments. **Conclusions**: These results underscore the potential of **NP@I-COSAN** as an effective boron delivery system for BNCT, offering a promising strategy to enhance boron accumulation within tumor cells and improve treatment efficacy.

## 1. Introduction

Nanoparticles (NPs) emerge as powerful tools in cancer treatment, offering targeted drug delivery and enhanced therapeutic efficacy [1,2]. Due to their small size and customizable surface properties, nanoparticles can be engineered to selectively accumulate in tumors, reducing side effects on healthy tissues. This targeted approach delivers higher drug concentrations directly to cancer cells, improving treatment outcomes. Additionally, nanoparticles can be designed to carry multiple therapeutic agents or imaging molecules, enabling treatment and real-time disease monitoring [3]. They are also used in advanced therapies like boron neutron capture therapy (BNCT) and hyperthermia, making them a promising frontier in cancer care [4,5].

Anionic boron cluster derivatives, especially cobaltabisdicarbollide (or **COSAN** ([Co(C_2_B_9_H_11_)_2_]^−^) exhibit a low nucleophilicity and charge density [6,7], along with hydrophobic properties [8,9] and remarkable chemical, thermal, and photochemical stability [10,11,12,13]. The intrinsic hydrophobicity of **COSAN** endows its protonated form and sodium salts with pronounced amphiphilicity, contributing to their high solubility in both aqueous and organic media [14,15]. This property also promotes their spontaneous organization into micelles and monolayer vesicles in aqueous environments [16,17,18,19,20]. Owing to these unique physicochemical attributes, **COSAN** is capable of permeating lipid bilayers and accumulating within various living cells [21], as evidenced by Raman [22,23] and fluorescence spectroscopy [24,25,26]. The iodine substitution in **I-COSAN** introduces a lipophilic character to the molecule, facilitating its interaction with lipid membranes and hydrophobic domains in biological systems [21]. This promotes its accumulation in biological systems, particularly in the cytoplasm [27]. These properties make **I-COSAN** an excellent candidate for cytoplasmic staining, drug delivery, and therapeutic applications. Radiolabeling of **COSAN** with a suitable radionuclide (^125^I and ^124^I) enables its visualization in vivo by Positron Emission Tomography (PET) [27,28]. **COSAN** derivatives have been covalently conjugated to a variety of macromolecules, including porphyrins and phthalocyanines [29,30,31,32], dendrimers [33,34], octasilsesquioxanes [35], and the surfaces of polymeric nanoparticles [36], gold nanoparticles [37], TiO_2_ [38], single-wall carbon nanotubes (SWCNTs) [39], and graphene oxide [27,40], resulting in materials with improved solubility, dispersibility, thermal stability, electrochemical properties, cellular uptake, and intracellular boron release. These distinctive features, combined with the high boron content, make this anion and its derivatives highly promising for potential medical applications as pharmacophores [41,42,43,44], particularly in BNCT [4,45,46,47,48].

BNCT is a targeted and versatile cancer treatment that utilizes the unique properties of boron atoms to destroy malignant tumor cells while selectively sparing healthy tissues [49,50,51]. The therapy relies on delivering a sufficient concentration of ^10^B atoms to the cancer cells (approximately 20–30 μg/g tumor) before exposing the patient to neutron radiation. Upon neutron capture, the ^10^B undergoes a nuclear reaction that results in localized cellular damage, effectively eliminating tumor cells while minimizing harm to surrounding normal tissues. A key challenge for successful BNCT has been finding boron carriers that achieve the necessary concentration and selectivity within tumors. Traditional boron-rich drugs have often failed due to insufficient targeting, leading to damage in healthy tissues. However, recent advances in nanotechnology offer a promising solution.

In recent years, boron cluster-based nanoparticles (NP@BC), including those featuring boron clusters like carboranes or metallacarboranes, have gained significant attention and have been applied to various fields, ranging from energy to biomedicine [37,52,53,54,55,56,57]. These NP@BC nanoparticles demonstrate exceptional biocompatibility and extended blood circulation times, rendering them promising platforms for detecting and treating various diseases. NP@BC are being explored for their potential in cancer therapies, especially in BNCT, as they present an ideal approach for this therapy; they can carry high concentrations of boron to tumors and can be engineered for enhanced stability and targeted uptake by cancer cells. Additionally, their versatility allows integration with imaging agents or other therapeutic strategies, creating a multifaceted and potent approach to cancer treatment through BNCT.

Herein, we combine the advantages of cobaltabisdicarbollide anions (**COSAN**) and specially designed nanoparticles (PolymP^®^-Link) to develop a potential therapeutic agent (**NP@I-COSAN**) with high boron loading for use in BNCT. The attachment of **I-COSAN** to the NP surface was confirmed through multiple characterization techniques, including attenuated total reflectance infrared (ATR-FTIR) spectroscopy, UV-Vis spectrophotometry, thermogravimetric analysis (TGA), and transmission electron microscopy/energy-dispersive X-ray spectroscopy (TEM/EDX). TEM images were also acquired from HeLa cells incubated with the functionalized nanoparticles, confirming excellent cellular internalization. In vitro cytotoxicity assays and in vivo evaluations using *C. elegans* as an animal model were performed to assess the biocompatibility of the synthesized hybrid nanoparticles. Additionally, intracellular boron uptake in HeLa cells was quantitatively measured using inductively coupled plasma mass spectrometry (ICP-MS). To further assess the efficacy of BNCT with **I-COSAN**-based nanoparticles (**NP@I-COSAN**), an in vitro proof-of-concept assay was performed, followed by an analysis of the cytotoxic effects using a colony-forming assay.

## 2. Results and Discussion

### 2.1. Synthesis and Characterization of NPs Decorated with I-COSAN (NP@I-COSAN)

In this study, the synthesis and characterization of **I-COSAN**-based nanoparticles (**NP@I-COSAN**) as a new boron carrier for BNCT were performed. The new hybrid (**NP@I-COSAN**) was synthesized by functionalizing commercial acrylic polymer-based nanoparticles **NPs** with the monoiodinated boron-cluster (**I-COSAN**, **1**). This cluster features a terminal amino group for reacting with functional groups of the NP’s surface and an iodine atom that provides lipophilicity, facilitating cell membrane penetration. Compound **1** was initially synthesized by introducing iodine crystals into a dichloromethane solution of [8-C_4_H_8_O_2_NH_3_-3,3′-Co(1,2-C_2_B_9_H_10_)(1′,2′-C_2_B_9_H_11_)], in accordance with previously established protocols [27]. Characterization by ATR-FTIR, ^1^H, ^11^B, and ^13^C NMR, and MALDI-TOF MS confirmed the formation of the pure compound.

**Scheme 1:**
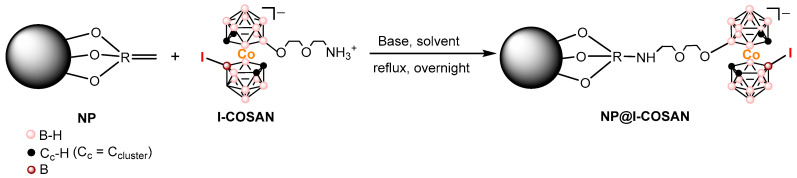
Representative scheme on the functionalization of **NPs** with **I-COSAN**, leading to the formation of **NP@I-COSAN**.

Covalent attachment of **I-COSAN** onto **NPs** was carried out using triethylamine (Et_3_N) in acetonitrile at reflux for 17 h under an inert atmosphere (N_2_). After this time, the solvent was evaporated, and EtOH was added, resulting in a light orange precipitate, which was confirmed as the expected product **NP@I-COSAN** (Scheme 1).

**NP@I-COSAN** was initially characterized by ATR-FTIR spectroscopy (Figure S1). The band at 2554 cm^−1^, attributed to υ(B-H, st), clearly confirms the grafting of compound **1** to the **NPs**.

The attachment of boron clusters onto the **NP** surface has been quantitatively determined by performing thermogravimetric analysis (TGA) under an air atmosphere and using a heating rate of 10 °C/min (Figure S2, Table S1). For **NPs**, the mass loss takes place around 220 °C, whereas in the case of **NP@I-COSAN**, it can be observed that the mass loss starts at a higher temperature, around 260 °C. In both cases, the mass loss is quite steep until 400 °C due to the removal of the oxygen-based functional groups of the **NPs** surface and the cleavage of the B_cluster_-O bond, respectively. The **NPs** sample was almost completely combusted at 500 °C, while there is a less pronounced mass loss for **NP@I-COSAN**, which is slowly combusted up to 800 °C. Regarding the starting **I-COSAN** material, we observe a similar behavior to that described before. Throughout the process, the carbon atoms from the NPs and the organic moiety of the boron-cluster compounds undergo complete oxidation.

Then, the degree of functionalization can be determined considering the residue left after complete combustion, as the carbon present in both **NPs** and the organic part of the boron derivative of all samples is oxidized. Therefore, the remaining material is considered to be the rest of the inorganic materials, such as oxidized boron clusters. As 14.29 wt% of residue was found in the **NP@I-COSAN** sample, this led us to a degree of functionalization of 253 µmol·g^−1^ (which means ca. 49 mg_B_·g^−1^).

Transmission electron microscopy (TEM) images (Figure S3) confirmed the NPs’ spherical shape after functionalization with **I-COSAN**.

UV-Vis analysis of dispersions of **NP@I-COSAN** was performed in H_2_O and EtOH (Figure S4). Nevertheless, the characteristic peak of **COSAN** at 446 nm appears to be masked by the nanoparticles’ absorption, whereas the peak at 200 nm, which also corresponds to COSAN, is visible in water but masked by the EtOH.

### 2.2. Cytotoxicity Studies

**Figure 1:**
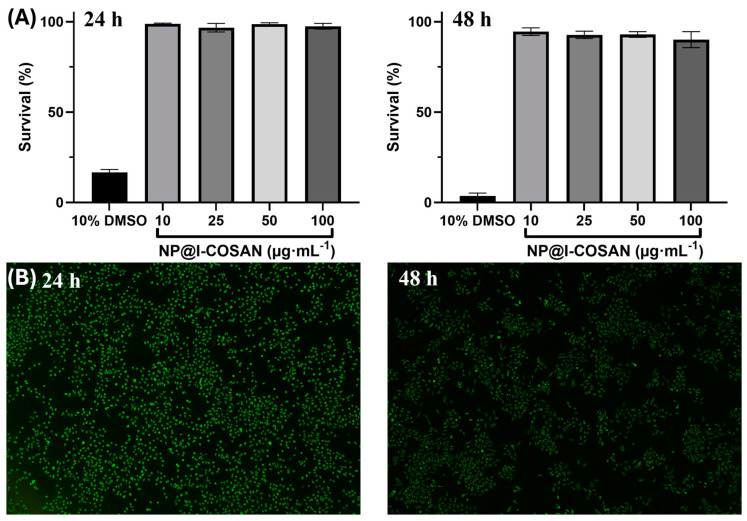
(**A**) Bar graphs indicating the percentage of HeLa cell survival after 24 h (left) and 48 h (right) of treatment with four different concentrations (10, 25, 50, and 100 μg∙mL^−1^) of **NP@I-COSAN** and the positive control (DMSO 10 vol. %), error bars mean ± 5%. (**B**) Fluorescence microscopy photographs of the LIVE/DEAD experiments of HeLa cells after being incubated for 24 h (left) and 48 h (right) with 100 μg∙mL^−1^ **NP@I-COSAN**.

The biocompatibility of **NP@I-COSAN** was evaluated in HeLa cells, following the same method used previously by our group for COSAN-functionalized graphene oxide (**GO-I-COSAN**) by a modified LDH assay [27], which avoids direct contact between the colorimetric indicator and the nanomaterial [58]. HeLa cells were incubated in 96-well plates for 24 and 48 h in the presence of **NP@I-COSAN** previously dispersed in DMSO (1% *v*/*v*) at the following four different concentrations: 10, 25, 50, and 100 μg∙mL^−1^. As controls, two additional samples were prepared, containing DMSO at 1% and 10% *v*/*v*, serving as the negative and positive references, respectively. After incubation, dead cells, including the living cells and NP@I-COSAN, were separated from the media. Therefore, the same protocol as that for **GO-I-COSAN** was followed to get a percentage of cell survival above 90% (Figure 1A) [27], both at 24 and 48 h of incubation for all the concentrations, which confirmed the low cytotoxicity of **NP@I-COSAN**. High cell mortality (>80%) after 24 h was observed in the positive control sample. At the same time, the negative control and the control containing 1% *v*/*v* DMSO showed similar cell viability values, thus confirming that this concentration of DMSO has no cell toxicity.

In addition to the modified LDH assay, LIVE/DEAD tests were also carried out for all the samples at both incubation times to confirm the non-cytotoxicity of the material. A green, fluorescent calcein marker for living cells and a red-fluorescence ethidium homodimer-1 (EthD-1) for dead cells were used for the staining procedure. Calcein and EthD-1 were excited at 485 nm and 530 nm, respectively, and fluorescence emissions were acquired at 530 nm and 645 nm, respectively. **NP@I-COSAN** showed very low cytotoxicity, as the majority of cells were alive even when they were treated with the maximum concentration of the hybrid for 48 h (Figure 1B). As expected, the positive control using 10% *v*/*v* DMSO showed a mortality rate higher than 80% after 48 h.

### 2.3. Cell Internalization of NP@I-COSAN

**Figure 2:**
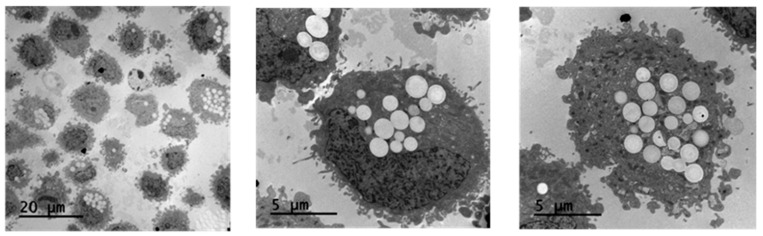
TEM images of the accumulation of **NP@I-COSAN** inside HeLa cells after 48 h of incubation with solutions of 0.05 M of **NP@I-COSAN**.

In vitro cytotoxicity studies have demonstrated that **NP@I-COSAN** is non-toxic to HeLa cells. The next step was to assess if our nanoparticles were effectively internalized by the cells. To address this, we investigated the cellular uptake of **NP@I-COSAN** and its effects on cellular ultrastructure. HeLa cells were incubated with **NP@I-COSAN** for 48 h and analyzed using TEM. TEM images confirmed the internalization of **NP@I-COSAN**, revealing abundant spherical nanoparticles within the cytoplasm (Figure 2). Notably, no nanoparticles were detected in the cell nucleus under the imaging conditions.

These observations align with previous studies on COSAN-based molecules and materials. Previous research using fluorescence confocal microscopy and TEM analysis demonstrated that COSAN derivatives can penetrate the cell membrane and accumulate in the cytoplasm [27,59,60]. This ability is attributed to the strong interaction between COSAN and proteins, which promotes its cytosolic retention and restricts diffusion through the nuclear envelope or nuclear pores. Similarly, TEM images further confirmed the presence of **NP@I-COSAN** within the cytoplasm of HeLa cells (Figure 2). Even more importantly, the presence of **NP@I-COSAN** in the cytoplasm did not alter cell size or morphology, suggesting that these nanoparticles’ composition and intracellular accumulation do not induce cytotoxicity, cellular inactivity, or tissue damage. These findings confirm that **NP@I-COSAN** nanoparticles are effectively internalized by cells without compromising cellular viability.

### 2.4. In Vivo Cytotoxicity Tests with C. elegans

**Figure 3:**
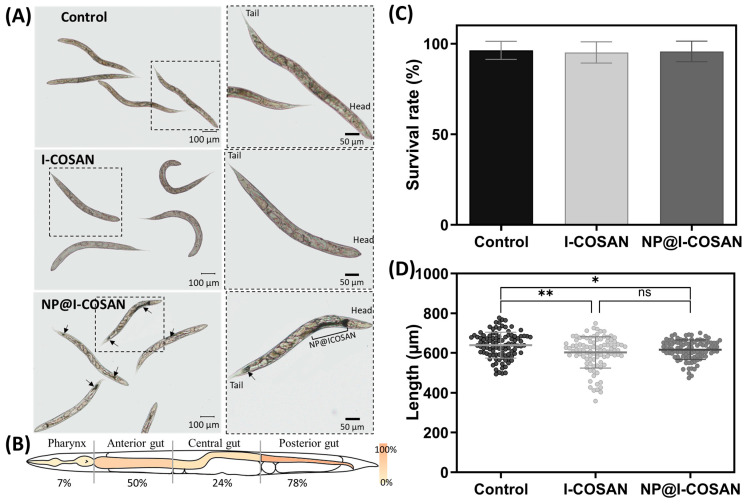
In vivo evaluation of **I-COSAN** and **NP@I-COSAN** in the *C. elegans* model. (**A**) Optical microscope images of control and **I-COSAN-** and **NP@I-COSAN**-treated worms. The right pictures are insets with a higher magnification than the left image. The worm’s head and tail are highlighted in the left inset. Arrows indicate the presence of **NP@I-COSAN**. The scale bar on the right is 100 µm, and the scale bar on the left is 50 µm. (**B**) Biodistribution of NP@I-COSAN. Percentage of worms with NP@I-COSAN in each of the four parts (pharynx, anterior gut, central gut, and posterior gut). Each region was represented with a gradient color from 0 to 100%. (**C**) Survival rate after 24 h of incubation with S-basal buffer (control) and 25 µg/mL **I-COSAN** and **NP@I-COSAN** (N = 3, n = 150). (**D**) The length of the worms was evaluated after 24 h of nanomaterial exposure (N = 3, n = 50). * *p* < 0.05, ** *p* < 0.01.

Following the in vitro evaluation of **NP@I-COSAN** and the confirmation of its non-toxicity toward mammalian cells, we proceeded with in vivo toxicity assessment using the small invertebrate model *Caenorhabditis elegans* (*C. elegans*). This model offers additional insights into the nanomaterial’s toxicological profile. We evaluated the biocompatibility of these nanomaterials in vivo using the 1 mm long nematode *C. elegans*. This nematode has been used as a model for decades due to its simplicity, straightforward maintenance, and elevated genetic homology with humans [61,62,63,64]. In addition, its transparency makes this model ideal for evaluating colored materials such as **NP@I-COSAN**. In this context, we exposed this worm synchronized in their L4 stage to 25 µg/mL of **I-COSAN** or **NP@I-COSAN** for 24 h. First, brownish clumps inside *C. elegans*’ intestinal tube were observed in **NP@I-COSAN**-exposed worms (Figure 3A), confirming the capacity of these worms to uptake the material. We measured the biodistribution of NP@I-COSAN along the worm gut, detecting that most of the nanoparticles were found at the anterior (50%) and posterior gut of *C. elegans* (78%) (Figure 3B). No visible damage was produced in worms by ingesting these nanomaterials. We did not detect the presence of **I-COSAN** molecules inside worms with the optical microscope, probably due to its light color. However, considering these molecules are smaller than the **NP@I-COSAN** complex, it is highly likely that worms also ingested them.

The biocompatibility of these nanomaterials was tested by measuring the worms’ survival rate and length after 24 h of exposure. The survival rate of **I-COSAN** and **NP@I-COSAN**-treated worms was similar to that of the control group (S-basal buffer) and close to 100% (Figure 3C). Regarding the length, worms exposed to **I-COSAN** and **NP@I-COSAN** were slightly shorter than control worms (ca. 6% and 4%, respectively) (Figure 3D). These results agree with the in vitro experiments and confirm the material’s biocompatibility in an entire organism, paving the way for their further evaluation in higher-complex organisms.

### 2.5. Boron Uptake in HeLa Cells Treated with ^10^B-L-Boronophenylalanine (BPA) and NP@I-COSAN

**Figure 4:**
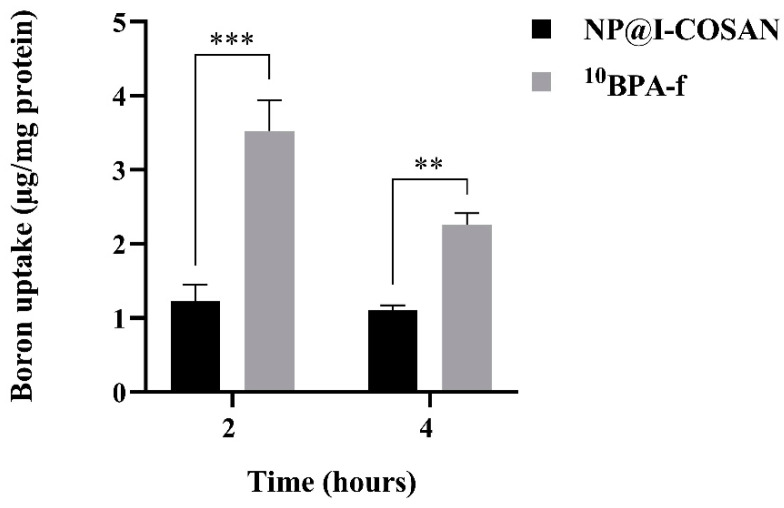
Boron uptake in HeLa cells was evaluated after exposure to 0.045 ppm of **^10^B**-**NP@I-COSAN** and 25 ppm of **^10^BPA-f** for 2 and 4 h. (**) *p* < 0.01 and (***) *p* < 0.001, according to the two-way ANOVA analysis (Tukey’s test).

For BNCT to be effective, the majority of boron atoms must be primarily intracellular rather than extracellular. When boron remains extracellular, the physical dose reaching the cell nuclei is limited to only 10% [65]. Thus, maximizing intracellular boron accumulation is crucial for achieving a therapeutically effective dose. Accordingly, we evaluated the intracellular boron levels in HeLa cells using inductively coupled plasma mass spectrometry (ICP-MS) to assess the potential of **NP@I-COSAN** as an antitumor agent in BNCT. Figure 4 illustrates the uptake of BPA-fructose conjugate enriched with boron-10 (**^10^BPA-f**), used as a reference compound, and **NP@I-COSAN** in the HeLa cell line at both time points. Although **^10^BPA-f** (25 ppm) exhibits a higher uptake, it is important to note that this assay used the same concentration of **NP@I-COSAN** (25 µg/mL) as in the in vivo assays with *C. elegans*, which corresponds to 0.045 ppm of **^10^B-NP@I-COSAN**. As a result, the amount of boron-10 used in the **^10^BPA-f** treatment was over 900 times higher than that in the **^10^B-NP@I-COSAN** treatment. Furthermore, after 4 h, **^10^BPA-f** uptake decreases to nearly half, likely due to an efflux mechanism [66], while the concentration of **^10^B-NP@I-COSAN** remains stable. This consistent retention is a significant advantage for future in vivo applications of BNCT, where maintaining compound presence within tumor cells or tissues is crucial.

### 2.6. In Vitro BNCT Proof of Concept Assay

**Figure 5:**
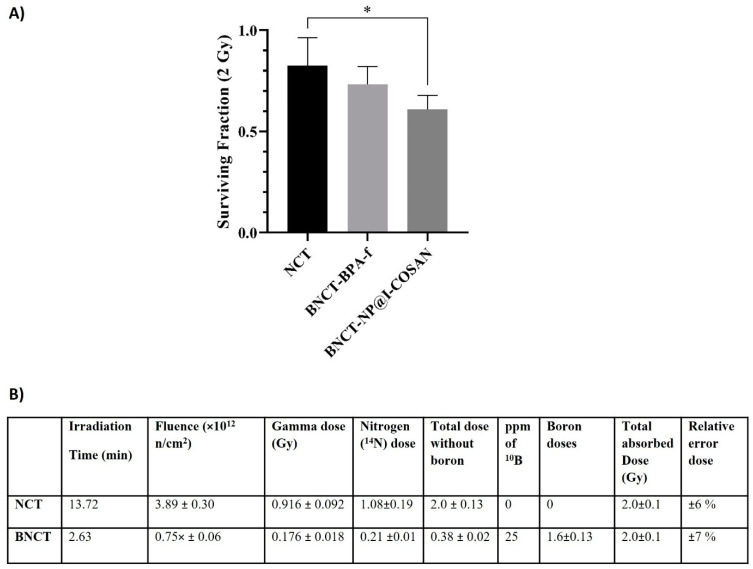
(**A**) Cell survival after treatment with 0.045 ppm of **^10^B**-**NP@I-COSAN** and 25 ppm of **^10^BPA-f**, post-neutron irradiation (2 Gy). (**B**) Dosimetry for thermal neutron irradiation without and with boron (25 ppm ^10^B). Note: Dosimetry for the BNCT groups is based on **BPA**-**f**, which has a boron-10 concentration of 25 ppm. (*) *p* < 0.05, according to the two-way ANOVA analysis (Tukey’s test).

**Figure 6:**
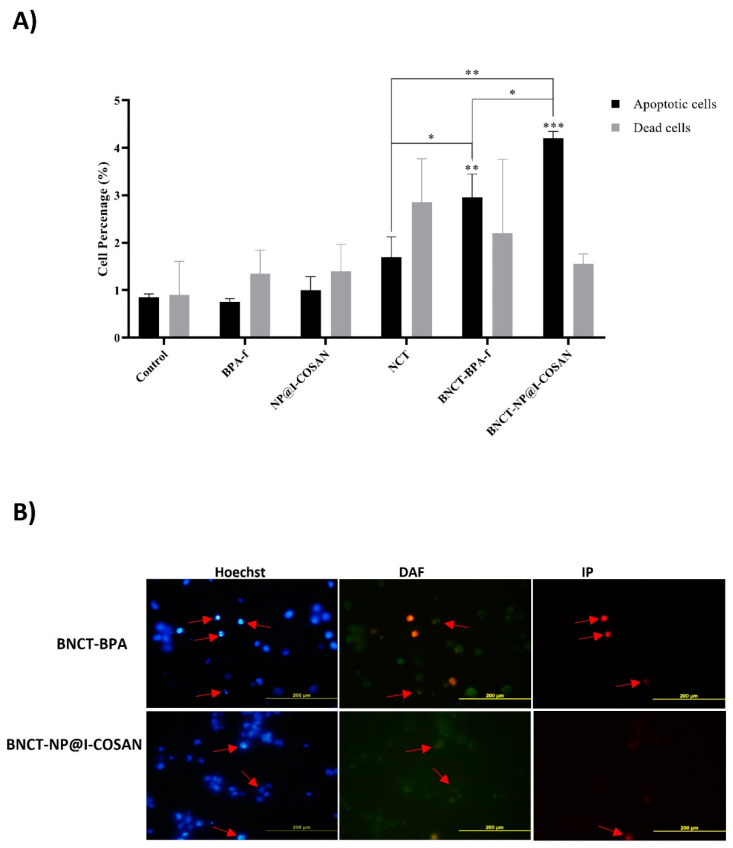
(**A**) Quantitative analysis of fluorescence microscopy at 48 h post-irradiation; (**B**) Post-irradiation analyses of apoptotic and necrotic events (dead cells) were performed using fluorescence microscopy. Representative images of stained cells were taken 48 h after irradiation. Apoptotic nuclei, indicated by Hoechst staining (red arrows), exhibited features such as peripheral chromatin condensation, cytoplasmic blebbing, and fragmentation. The cytoplasm of viable cells was labeled with 4,5-diaminofluorescein (DAF), whereas necrotic cells were identified using propidium iodide (IP) staining (red arrows). (*) *p* < 0.05, (**) *p* < 0,01, (***) *p* < 0.001, according to the two-way ANOVA analysis (Tukey’s test).

The efficacy of **^10^B-NP@I-COSAN** as a boron neutron capture therapy agent was systematically assessed in the HeLa cell line (Figure 5). Cells were incubated with either 0.045 ppm of **^10^B-NP@I-COSAN** or 25 ppm of ^10^B of ^10^**BPA-f** (2.31 mM) for 2 h. Following incubation, the cells were irradiated with a neutron flux of 5 × 10^9^ n/ cm²·s, delivering a dose of 2 Gy and a fluence of 3.89 × 10^12^ neutrons cm^−2^, as determined by the dosimetric studies in Figure 6B. Cytotoxicity from neutron capture reactions was measured using the MTT (3-(4,5-dimethylthiazol-2-yl)-2,5 diphenyltetrazolium bromide) assay. Figure 6A illustrates a statistically significant difference in survival fractions between cells treated with **^10^B-NP@I-COSAN** and those exposed solely to the neutron beam. However, no significant difference in survival was observed between the **^10^B-NP@I-COSAN** and **^10^BPA-f** treatments. It is important to emphasize that the concentration of boron-10 in **^10^BPA-f** was almost 900 times greater than that in **^10^B-NP@I-COSAN**.

### 2.7. Study of the Cell Death Mechanism

This study investigated the mechanisms of cell death induced by **^10^BPA-f** and **^10^B**-**NP@I-COSAN** within the context of the BNCT (Figure 6). The evaluation of these mechanisms was carried out at a dose of 2 Gy, 48 h post-neutron irradiation, along with a concentration of 0.045 ppm of **^10^B-NP@I-COSAN** and 25 ppm of **^10^BPA-f**. The effect of both treatments on HeLa cell death levels was analyzed using fluorescence microscopy. The results demonstrate a marked increase in cell death through apoptosis and necrosis across all treatments involving neutron activation. Remarkably, when HeLa cells were exposed to neutron capture treatment (NCT) alone, they displayed necrosis. In contrast, exposure to boron neutron capture therapy with **^10^BPA-f** or **^10^B**-**NP@I-COSAN** resulted in an increased number of apoptotic cells. This finding suggests that incorporating boron compounds enhances the efficacy of cell death mechanisms compared to NCT alone. Furthermore, compared to the **^10^BPA-f** and NCT-only groups, treatment with BNCT-**^10^B**-**NP@I-COSAN** resulted in a statistically significant increase in apoptotic cell death. These findings underscore the critical role of boron delivery systems in BNCT and highlight the enhanced apoptotic response associated with **^10^B**-**NP@I-COSAN**. Future research will aim to elucidate the mechanisms underlying this enhanced response and explore the potential clinical applications of these results in cancer treatment.

## 3. Materials and Methods

**Characterization.** ATR-FTIR spectra were recorded on a JASCO FT/IR-4700 (Madrid, Spain) spectrometer at high resolution. Samples were prepared by drop-drying sample dispersions on 2-propanol onto a preheated hot plate at 80 °C. TGA analyses were performed under flowing air at a heating rate of 10 °C min^−1^ up to 900 °C using a Jupiter (Netzsch Instrument, Selb, Germany) thermogravimeter analyzer. TEM images were performed using a transmission electron microscope (TEM) 120 KV JEOL 1210 (Jeol Ltd., Peabody, MA, USA). The UV-Vis spectra were recorded on a JASCO V-780 UV-Visible/NIR (Madrid, Spain) spectrophotometer, using spectroscopic grade EtOH and H_2_O (Sigma-Aldrich, St. Louis, MO, USA), in a normal quartz cuvette having a 1 cm path length.

**Materials.** All reactions were performed under an atmosphere of dinitrogen employing standard Schlenk techniques. For this study, [8-C_4_H_8_O_2_NH_3_-3,3′-Co(1,2-C_2_B_9_H_10_)(1′,2′-C_2_B_9_H_11_)] was obtained as described in the literature [40,67]. Moreover, [8-C_4_H_8_O_2_NH_3_-3,3′-Co(1,2-C_2_B_9_H_10_)(8′-I-1′,2′-C_2_B_9_H_11_)] was synthesized according to the methods described in the literature [27]. PolymP^®^-Link nanoparticles were purchased from the NanoMyP^®^ company (Granada, Spain).

**Synthesis of nanoparticles NP@I-COSAN or NP@[8-C_4_H_8_O_2_NH_3_-3,3′-Co(1,2-C_2_B_9_H_10_)(8′-I-1′,2′-C_2_B_9_H_11_)]. NPs** (25 mg) and the compound **I-COSAN** (50 mg, 0.090 mmol) were dried for 2 h at 100 °C under vacuum. Then, 2 mL of dry Et_3_N and 5 mL of acetonitrile were added, and the heterogeneous mixture was stirred for 17 h at reflux under a nitrogen atmosphere. After this time, the solvent was removed under vacuum, and the residue was washed with EtOH (4 mL), sonicated, and filtered off through a 0.2 μm polycarbonate membrane (Millipore, Burlington, MA, USA) and washed again with EtOH. The sample was then dried to obtain **NP@I-COSAN** as an orange solid. FT-IR of 2944 (m, C-H st), 2554 (w, B-H st), and 1720 (s, C-O st).

### 3.1. Cytotoxicity Assays

Four distinct solid samples of **NP@I-COSAN** were prepared, each corresponding to concentrations of 10, 25, 50, and 100 μg∙mL^−1^. The samples were sterilized by overnight UV germicidal irradiation and subsequently dissolved in DMSO (to a final concentration of 1%). After a brief sonication for 1 min, each sample was diluted in culture medium (MEM-alpha, supplemented with 10% fetal bovine serum and Penicillin-Streptomycin-Fungizone solution from GIBCO, Waltham, MA USA). The samples were then subjected to sonication for an additional hour to ensure proper dispersion.

For the biological assays, HeLa cells (ATCC CCL-2) were used. These cells were cultured in MEM-alpha medium, supplemented with 10% fetal bovine serum and 4 mM L-glutamine at 37 °C in a 5% CO_2_ atmosphere.

Cytotoxicity of **NP@I-COSAN** was assessed at the following two time points: 24 and 48 h. HeLa cells were seeded into 96-well plates at densities of 3500 cells per well for the 24 h time point and 1800 cells per well for the 48 h time point, with 100 μL of culture medium per well. After a 24 h incubation to allow cell attachment, 100 μL of each **NP@I-COSAN** concentration was added to the corresponding wells, followed by further incubation for either 24 or 48 h. The effect of **NP@I-COSAN** on cell viability was determined using a modified LDH assay (CytoTox 96 Non-Radioactive Cytotoxicity Assay, Promega, Madison, WI, USA), as described by Ali Boucetta et al. [58]. Additionally, cell viability was evaluated using a live/dead viability/cytotoxicity kit (Thermo Fisher, Barcelona, Spain)), which is based on cellular esterase activity and membrane integrity. HeLa cells were seeded into 24-well plates at 10,700 cells per well for 24 h and 20,700 cells per well for 48 h. Live cells were stained green, and images were captured using a Nikon inverted fluorescence microscope (Nikon Instruments Inc., Long Beach, CA, USA).

### 3.2. TEM of HeLa Cells

For sample preparation, HeLa cells (CCL-2) p.X+17, obtained from the American Type Culture Collection, were employed. The medium used for cell seeding consisted of MEM-alpha supplemented with 10% fetal bovine serum (1), while the assay medium (2) contained a combination of penicillin, streptomycin, and fungizone to prevent microbial contamination. On the first day, HeLa cells were seeded in three 75 cm^2^ T-flasks, each containing 15 mL of MEM-alpha medium (1). Meanwhile, **NP@I-COSAN** (0.5 mg, in duplicate) was sterilized overnight under a UV fluorescent lamp inside a laminar flow hood. Subsequently, 100 μL of DMSO (1 vol %) was added to the **NP@I-COSAN**, followed by a brief vortex mixing and a one-minute sonication. The prepared dispersions were then transferred into sterilized test tubes containing 10 mL of fresh assay medium (2) and subjected to an additional hour of sonication. Afterward, the original culture medium (1) was removed from the HeLa cells, and the **NP@I-COSAN** dispersions in assay medium (2) were added to the cultures in duplicate. A 10 mL control solution was prepared under the same conditions. The three samples—two **NP@I-COSAN** replicates and the control—were incubated at 37 °C in a humidified atmosphere with 5% CO_2_ for 48 h. Following incubation, cells were washed twice with PBS and detached using trypsin. The collected pellets were transferred into test tubes and subjected to centrifugation at 1500 rpm for 6 min in PBS. Subsequently, the cells were fixed in a 4% formaldehyde solution prepared in PBS for 15 min at ambient temperature. After fixation, the pellets were resuspended in the same solution and placed into pre-rinsed Eppendorf tubes (2 mL PBS) and then stored at 4 °C. The samples were gradually dehydrated through an ascending ethanol series and treated with propylene oxide before being embedded in Durcupan resin (Fluka AG, Buchs, Switzerland). A Power-Tome MT-XL ultramicrotome (RMC/Sorvall, Tucson, AZ, USA) was used to cut thin sections of 60 nm in thickness, which were then placed on copper grids coated with Epon and Spur resins. These sections were then stained with lead citrate [68] and visualized under a Jeol 1400 transmission electron microscope (Jeol Ltd., MA, USA.), operating at 80 kV and fitted with a Gatan Orius SC200 CCD camera (Gatan Inc., Pleasanton, CA, USA). Representative images of distinct fields were acquired at magnifications ranging from 100,000× to 600,000× for each sample and technique.

### 3.3. C. elegans In Vivo Evaluation

*C. elegans* Bristol strain N1 and *Escherichia coli* OP50 were purchased from the Caenorhabditis Genetic Center (Church St SE, Minnesota, USA). Using standard procedures [69], worms were cultured at 20 °C [70]. Synchronized L4 worms were treated separately with S-basal buffer (control), 25 μg·mL^–1^ of **I-COSAN**, and 25 μg·mL^–1^ of **NP@I-COSAN** in a final volume of 100 μL for 24 h in 96-well plates. **I-COSAN** and **NP@I-COSAN** were dissolved in S-basal buffer and added to each well to obtain the concentration mentioned above. Approximately 9 ± 3 adult worms (N = 3, n = 150) were distributed per well. Survival rate was counted by tapping the plate and counting worms that moved as alive [52]. Worms were washed off the material by centrifuge steps of 1 min at 4400 rpm. The supernatant was discarded, and fresh S-basal was added. Then, worms were fixed with paraformaldehyde (1%) and mounted onto a microscope slide to obtain microscope images with an Olympus BX51 (Barcelona, Spain). For the biodistribution assay, the alimentary system of worms was divided into four segments: pharynx, anterior gut, central gut, and posterior gut. Results were expressed as a percentage of worms with NP@I-COSAN in each part (n = 54). Length was measured using Fiji ImageJ software (https://imagej.net/software/fiji/downloads, accessed on 15 September 2022) (N = 3, n = 50). Both assays, survival and length, were performed in triplicate. For statistical analysis, the Kruskal–Wallis test with Dunn’s post-correction was performed using GraphPad Prism, version 9.

### 3.4. Determination of Intracellular Boron by Inductively Coupled Plasma Mass Spectrometry (ICP-MS)

Exponentially growing cells were incubated with **NP@I-COSAN** (0.1 μM, dissolved in DMSO) or **^10^B-BPA-f** (2.31 mM, diluting the stock solution to a final volume of 500 μL with water). The stock solution of **^10^B-BPA-f** was prepared at a concentration of 30 mg as follows: **^10^B-BPA-f** per mL (0.14 M) as B-BPA was combined in water with a 10% molar excess of fructose, adjusting the pH to 9.5−10.0 with an aqueous solution of NaOH, and the resulting mixture was stirred until all solids dissolved; finally, the pH was readjusted to 7.4 with an aqueous solution of HCl. After incubation (1, 2, and 4 h), the cells were washed twice with 1× PBS at 4 °C. The pellets were digested with formic acid (500 μL). An aliquot of 250 μL was diluted to 1.0 mL with an aqueous solution containing 1 mg/L of Y (0.75 mL) as internal standards. The boron uptake was measured by ICP-MS Perkin-Elmer, model ELAN-DRC II. The internal standard used was ^6^Li. Matrix-matched standard solutions containing the internal standard elements and ^10^B between 0.5 and 100 µg/mL were employed for daily calibration. An internal measurement control was also used, with each certain number of samples adding a sample of known concentration (QC). Each sample was diluted to half. The other aliquot of 250 μL was dried and resuspended in an aqueous solution of NaOH (0.3 N), and total proteins were determined by the Lowry method. The boron amount was referred to the total proteins.

### 3.5. Neutron-Irradiation Procedures

Cells were irradiated in the thermal column of the RA-3 reactor, a 9 MW nuclear facility located in Ezeiza, Argentina, which provides a highly thermalized and homogeneous irradiation field. The thermal neutron flux was approximately (1.0 ± 0.1) × 10^10^ neutrons cm^−2^ s^−1^, with a cadmium ratio of 4100 for gold foils, thereby allowing the neglect of contributions from fast neutron doses. The gamma dose rate was estimated to be around (6.0 ± 0.2) Gy h^−1^. The total absorbed dose was calculated by summing the contributions from photon exposure, nitrogen capture (assuming a nitrogen content of 3.5% by weight), and boron capture. Prior to each irradiation, the neutron flux at the irradiation site was verified using calibrated Rh-SPND detectors configured in a manner representative of the experimental setup (96-well plates or 60 cm plates without cells). Concurrently, a boron-coated ionization chamber provided monitoring of the neutron signal. Irradiation times were then determined to ensure a delivery of 2 Gy, with an estimated uncertainty of 10%. Comprehensive dosimetric assessments were conducted for each treatment, encompassing time, fluence, and the contributions of each component to the total absorbed dose from the neutron beam, both with and without boron. For dosimetric calculations, the intracellular concentration of boron at the time of irradiation was assumed to be uniformly distributed both inside and outside the cells.

### 3.6. Irradiation of **NP@I-COSAN** or **^10^BPA-f**-Treated HeLa Cells Assays

HeLa cells were plated at a density of 2500 cells per well in 96-well plates, with eight wells per treatment group. The cells were subsequently exposed to treatments with **NP@I-COSAN** (25 µg/mL dissolved in DMSO, corresponding to 0.045 ppm boron) or **^10^BPA-f** (2.31 mM dissolved in water, corresponding to 25 ppm boron) for 1 h post-plating. Irradiated DMSO (5%) or irradiated water-treated cells, along with non-irradiated compound-treated cells, were used as control groups. After this incubation, the cells were exposed to thermal neutron irradiation with a total fluence of 3.88 × 10^12^ ± 3 × 10^11^ neutrons/cm^2^.

### 3.7. Cell Surviving Assay

After irradiation, the culture medium was replaced, and the cells were incubated at 37 °C for 7 days. Subsequently, 20 μL of the vital dye MTT (Sigma 128, 0.5%, *w*/*v* in PBS) was added to the culture medium. After a 4 h incubation at 37 °C, absorbance was measured at 540 nm. The results are presented as the survival fraction relative to the untreated controls.

### 3.8. Frequency of Cell Death Analysis

Cell death of irradiated treated B cells (25 or 0.045 ppm) was assessed morphologically by fluorescence microscopy (Olympus BX51, 40×, Tokyo, Japan) at 48 h post-irradiation. Briefly, cell pellets (10^6^) were resuspended in 100 μL of a staining solution containing PI, DAF, and Hoechst 33258 at concentrations of 0.6, 0.1, and 0.6 mg/mL, respectively. Results are expressed as a percentage of living cells, apoptotic cells, or necrotic cells per 200 total cells.

## 4. Conclusions

COSAN-functionalized nanoparticles (**NP@I-COSAN**) were successfully synthesized and characterized using advanced analytical techniques. The attachment of monoiodinated boron clusters (**I-COSAN**) to commercial polymer-based nanoparticles was confirmed, ensuring high boron loading and stability. In vitro and in vivo studies demonstrated the biocompatibility of **NP@I-COSAN**. Cytotoxicity assays in HeLa cells showed high cell survival rates, while in vivo evaluations using *C. elegans* model confirmed the absence of adverse effects, paving the way for their application in BNCT. TEM analysis revealed efficient internalization of **NP@I-COSAN** in HeLa cells, with nanoparticles predominantly localized in the cytoplasm. This cellular uptake is critical for the efficacy of boron delivery in BNCT. Quantitative ICP-MS analysis highlighted the superior boron retention delivered by **NP@I-COSAN** in cells compared to conventional agents like **^10^BPA-f**. This stability is advantageous for ensuring consistent boron availability during neutron irradiation. An in vitro proof-of-concept BNCT assay demonstrated that **NP@I-COSAN** effectively induced tumor cell death via apoptosis at significantly lower boron concentrations than traditional boron delivery systems. The observed apoptotic response underscores the enhanced therapeutic potential of **NP@I-COSAN**. The study establishes **NP@I-COSAN** as a promising boron delivery system for BNCT.

## Data Availability

The original contributions presented in this study are included in the article/Supplementary Material. Further inquiries can be directed to the corresponding authors.

## Supplementary Materials

The following supporting information can be downloaded at: https://www.mdpi.com/article/10.3390/ph18040466/s1. Figure S1. FT-IR spectrum of **NP@-I-COSAN**; Figure S2. TGA of **NP@I-COSAN** (continuous line) and the starting compounds NPs (dashed line) and **I-COSAN** (dotted line). TGA were performed under flowing air at a heating rate of 10 °C∙min^−1^; Figure S3. TEM images and EDX analysis of **NP@I-COSAN**; Figure S4: UV-vis spectra of **NP@-ICOSAN** in EtOH and H_2_O; Table S1. Selected TGA data.

## References

[1] Gavas S., Quazi S., Karpiński T.M. (2021). Nanoparticles for Cancer Therapy: Current Progress and Challenges. Nanoscale Res. Lett..

[2] Al-Thani A.N., Jan A.G., Abbas M., Geetha M., Sadasivuni K.K. (2024). Nanoparticles in cancer theragnostic and drug delivery: A comprehensive review. Life Sci..

[3] Cheng Z., Li M., Dey R., Chen Y. (2021). Nanomaterials for cancer therapy: Current progress and perspectives. J. Hematol. Oncol..

[4] Barth R.F., Vicente M.G., Harling O.K., Kiger W.S., Riley K.J., Binns P.J., Wagner F.M., Suzuki M., Aihara T., Kato I. (2012). Current status of boron neutron capture therapy of high grade gliomas and recurrent head and neck cancer. Radiat. Oncol..

[5] Marforio T.D., Carboni A., Calvaresi M. (2023). In Vivo Application of Carboranes for Boron Neutron Capture Therapy (BNCT): Structure, Formulation and Analytical Methods for Detection. Cancers.

[6] Masalles C., Borrós S., Viñas C., Teixidor F. (2000). Are Low-Coordinating Anions of Interest as Doping Agents in Organic Conducting Polymers?. Adv. Mater..

[7] Masalles C., Llop J., Viñas C., Teixidor F. (2002). Extraordinary Overoxidation Resistance Increase in Self-Doped Polypyrroles by Using Non-conventional Low Charge-Density Anions. Adv. Mater..

[8] Rak J., Dejlová B., Lampová H., Kaplánek R., Matějíček P., Cígler P., Král V. (2013). On the Solubility and Lipophilicity of Metallacarborane Pharmacophores. Mol. Pharm..

[9] Dąbrowska A., Matuszewski M., Zwoliński K., Ignaczak A., Olejniczak A.B. (2018). Insight into lipophilicity of deoxyribonucleoside-boron cluster conjugates. Eur. J. Pharm. Sci..

[10] Grimes R.N. (2016). Carboranes.

[11] Housecroft C.E. (2011). Boron: Metallacarbaboranes. Encyclopedia of Inorganic and Bioinorganic Chemistry.

[12] Sivaev I.B., Bregadze V.I. (1999). Chemistry of Cobalt Bis(dicarbollides). A Review. Collect. Czech. Chem. Commun..

[13] Cabrera-González J., Sánchez-Arderiu V., Viñas C., Parella T., Teixidor F., Núñez R. (2016). Redox-Active Metallacarborane-Decorated Octasilsesquioxanes. Electrochemical and Thermal Properties. Inorg. Chem..

[14] Viñas C., Tarrés M., González-Cardoso P., Farràs P., Bauduin P., Teixidor F. (2014). Surfactant Behaviour of Metallacarboranes. A Study Based on the Electrolysis of Water. Dalton Trans..

[15] Gassin P.-M., Girard L., Martin-Gassin G., Brusselle D., Jonchère A., Diat O., Viñas C., Teixidor F., Bauduin P. (2015). Surface Activity and Molecular Organization of Metallacarboranes at the Air-Water Interface Revealed by Nonlinear Optics. Langmuir.

[16] Bauduin P., Prevost S., Farràs P., Teixidor F., Diat O., Zemb T. (2011). A Theta-Shaped Amphiphilic Cobaltabisdicarbollide Anion: Transition from Monolayer Vesicles to Micelles. Angew. Chem. Int. Ed..

[17] Brusselle D., Bauduin P., Girard L., Zaulet A., Viñas C., Teixidor F., Ly I., Diat O. (2013). Lyotropic Lamellar Phase Formed from Monolayered θ-Shaped Carborane-Cage Amphiphiles. Angew. Chem. Int. Ed..

[18] Uchman M., Ďorďovič V., Tošner Z., Matějíček P. (2015). Classical Amphiphilic Behavior of Nonclassical Amphiphiles: A Comparison of Metallacarborane Self-Assembly with SDS Micellization. Angew. Chem. Int. Ed..

[19] Fernández-Álvarez R., Ďorďovič V., Uchman M., Matějíček P. (2018). Amphiphiles without Head-and-Tail Design: Nanostructures Based on the Self-Assembly of Anionic Boron Cluster Compounds. Langmuir.

[20] Malaspina D.C., Viñas C., Teixidor F., Faraudo J. (2020). Atomistic Simulations of COSAN: Amphiphiles without a Head-and-Tail Design Display “Head and Tail” Surfactant Behavior. Angew. Chem. Int. Ed..

[21] Tarrés M., Canetta E., Viñas C., Teixidor F., Harwood A.J. (2014). Imaging in living cells using νB–H Raman spectroscopy: Monitoring COSAN uptake. Chem. Commun..

[22] Tarrés M., . Canetta E., Paul E., Forbes J., Azzouni K., Viñas C., Teixidor F., Harwood A. (2015). Biological Interaction of Living Cells with COSAN-Based Synthetic Vesicles. Sci. Rep..

[23] Alamón B.C., Dávila B., García M.F., Nievas S., Dagrosa M.A., Thorp S., Kovacs M., Trias E., Faccio R., Gabay M. (2023). A Potential Boron Neutron Capture Therapy Agent Selectively Suppresses High-Grade Glioma: In Vitro and in Vivo Exploration. Mol. Pharm..

[24] Uchman M., Jurkiewicz P., Cígler P., Grüner B., Hof M., Procházka K., Matějíček P. (2010). Interaction of Fluorescently Substituted Metallacarboranes with Cyclodextrins and Phospholipid Bilayers: Fluorescence and Light Scattering Study. Langmuir.

[25] Chaari M., Gaztelumendi N., Cabrera-González J., Peixoto-Moledo P., Viñas C., Xochitiotzi-Flores E., Farfán N., Ben Salah A., Nogués C., Núñez R. (2018). Fluorescent BODIPY-Anionic Boron Cluster Conjugates as Potential Agents for Cell Tracking. Bioconjugate Chem..

[26] Cabrera-González J., Muñoz Flores B.M., Viñas C., Chávez-Reyes A., Dias H.V.R., Jiménez Pérez V.M., Nuñez R. (2018). Organotin Dyes Bearing Anionic Boron Clusters as Cell-Staining Fluorescent Probes. Chem. Eur. J..

[27] Ferrer-Ugalde A., Sandoval S., Pulagam K.R., Muñoz-Juan A., Laromaine A., Llop J., Tobias G., Núñez G. (2021). Radiolabeled cobaltabis (dicarbollide) anion–graphene oxide nanocomposites for in vivo bioimaging and boron delivery. ACS Appl. Nano Mater..

[28] Gona K.B., Zaulet A., Gómez-Vallejo V., Teixidor F., Llop J., Viñas C. (2014). COSAN as a molecular imaging platform: Synthesis and “in vivo” imaging. Chem. Commun..

[29] Hao E., Vicente M.G.H. (2005). Expeditious synthesis of porphyrin–cobaltacarborane conjugates. Chem. Commun..

[30] Hao E., Sibrian-Vázquez M., Serem W., Garno J.C., Fronczek F.R., Vicente M.G.H. (2007). Synthesis, aggregation and cellular investigations of porphyrin-cobaltacarborane conjugates. Chem. Eur. J..

[31] Hao E., Zhang M., Wenbo E., Kadish K.M., Froczeck F.R., Courtney B.H., Vicente M.G.H. (2008). Synthesis and Spectroelectrochemistry of N-Cobaltacarborane Porphyrin Conjugates. Bioconjugate Chem..

[32] Atmaca G.Y., Dizman C., Eren T., Erdogmus A. (2015). Novel axially carborane-cage substituted silicon phthalocyanine photosensitizer; synthesis, characterization and photophysicochemical properties. Spectrochim. Acta Part A Mol. Biomol. Spectrosc..

[33] Juárez-Pérez E.J., Viñas C., Teixidor F., Núñez R. (2009). Polyanionic carbosilane and carbosiloxane metallodendrimers based on cobaltabisdicarbollide derivatives. Organometallics.

[34] Núñez R., Juárez-Pérez E.J., Teixidor F., Santillan R., Farfán N., Abreu A., Yépez R., Viñas C. (2010). Decorating Poly(alkyl aryl-ether) Dendrimers with Metallacarboranes. Inorg. Chem..

[35] Cabrera-González J., Chaari M., Teixidor F., Viñas C., Núñez R. (2020). Blue Emitting Star-Shaped and Octasilsesquioxane-Based Polyanions Bearing Boron Clusters. Photophysical and Thermal Properties. Molecules.

[36] Ďorďovič V., Uchman M., Reza M., Ruokolainen J., Zhigunov A., Ivankov O.I., Matejicek P. (2016). Cation-sensitive compartmentalization in metallacarborane containing polymer nanoparticles. RSC Adv..

[37] Pulagam K.R., Gona K.B., Gómez-Vallejo V., Meijer J., Zilberfain C., Estrela-Lopis I., Baz Z., Cossío U., Llop J. (2019). Gold Nanoparticles as Boron Carriers for Boron Neutron Capture Therapy: Synthesis, Radiolabelling and In Vivo Evaluation. Molecules.

[38] Juárez-Pérez E.J., Mutin P.H., Granier M., Teixidor F., Núñez R. (2010). Anchoring of phosphorus-containing cobaltabisdicarbollide derivatives to titania surface. Langmuir.

[39] Cabana L., González-Campo A., Ke X., Van Tendeloo G., Núñez R., Tobias G. (2015). Efficient Chemical Modification of Carbon Nanotubes with Metallacarboranes. Chem. Eur. J..

[40] Cabrera-González J., Cabana L., Ballesteros B., Tobias G., Núñez R. (2016). Highly Dispersible and Stable Anionic Boron Cluster–Graphene Oxide Nanohybrids. Chem. Eur. J..

[41] Plešek J. (1992). Potential applications of the boron cluster compounds. Chem. Rev..

[42] Viñas C. (2013). The uniqueness of boron as a novel challenging element for drugs in pharmacology, medicine and for smart biomaterials. Future Med. Chem..

[43] Leśnikowski Z.J. (2016). Challenges and Opportunities for the Application of Boron Clusters in Drug Design. J. Med. Chem..

[44] Scholz M., Hey-Hawkins E. (2011). Carbaboranes as Pharmacophores: Properties, Synthesis, and Application Strategies. Chem. Rev..

[45] Sivaev I.B., Bregadze V.V. (2009). Polyhedral Boranes for Medical Applications: Current Status and Perspectives. Eur. J. Inorg. Chem..

[46] Sibrian-Vázquez M., Vicente M.G.H., Hosmane N.S. (2012). Boron Tumor-Delivery for BNCT: Recent Developments and Perspectives. Boron Science: New Technologies and Applications.

[47] Fuentes I., Garcia-Mendiola T., Sato S., Pita M., Nakamura H., Lorenzo E., Teixidor F., Marques F., Viñas C. (2018). Metallacarboranes on the Road to Anticancer Therapies: Cellular Uptake, DNA Interaction, and Biological Evaluation of Cobaltabisdicarbollide [COSAN]^−^. Chem. Eur. J..

[48] Couto M., Cerecetto H. (2024). Advancements in the Synthesis and Biological Properties of Carboranes and High-Boron Related Compounds: A Comprehensive Exploration with Emphasis on BNCT Applications. J. Braz. Chem. Soc..

[49] Xuan S., Vicente M.G.H., Hey-Hawkins E., Viñas C. (2018). Recent Advances in Boron Delivery Agents for Boron Neutron Capture Therapy (BNCT). Boron-based Compounds: Potential and Emerging Applications in Biomedicine.

[50] Barth R.F., Mi P., Yang W. (2018). A realistic appraisal of boron neutron capture therapy as a cancer treatment modality. Cancer Commun..

[51] Hu K., Yang Z., Zhang L., Xie L., Wang L., Xu H., Josephson L., Liang S.H., Zhang M.-R. (2020). Boron agents for neutron capture therapy. Coord. Chem. Rev..

[52] Oleshkevich E., Morancho A., Saha A., Galenkamp K.M.O., Grayston A., Gennatti Crich S., Alberti D., Protti N., Comella J.X., Teixidor F. (2019). Combining Magnetic Nanoparticles and Icosahedral Boron Clusters in Biocompatible Inorganic Nanohybrids for Cancer Therapy. Nanomed. Nanotechnol. Biol. Med..

[53] Ciani L., Bortolussi S., Postum I., Cansolino L., Ferrari C., Panza L., Altieri S., Ristori S. (2013). Rational design of gold nanoparticles functionalized with carboranes for application in Boron Neutron Capture. Intern. J. Pharm..

[54] Xiong H., Zhou D., Qi Y., Zhang Z., Xie Z., Chen X., Jing X., Meng F., Huang Y. (2015). Doxorubicin-Loaded Carborane-Conjugated Polymeric Nanoparticles as Delivery System for Combination Cancer Therapy. Biomacromolecules.

[55] Wang Y., Xu Y., Yang J., Qiu X., Li N., Zhu Y., Yan L., Li W., Huang X., Liang K. (2021). Carborane Based Mesoporous Nanoparticles as a Potential Agent for BNCT. Mater. Chem. Front..

[56] Zhu Y., Lin Y., Zhu Y.Z., Lu J., Maguire J.A., Hosmane N.S. (2010). Boron Drug Delivery via Encapsulated Magnetic Nanocomposites: A New Approach for BNCT in Cancer Treatment. J. Nanomater..

[57] Grzelczak M.P., Danks S.P., Klipp R.C., Belic D., Zaulet A., Kunstmann-Olsen C., Bradley D.F., Tsukuda T., Viñas C., Teixidor F. (2017). Ion Transport across Biological Membranes by Carborane-Capped Gold Nanoparticles. ACS Nano.

[58] Ali-Boucetta H., Al-Jamal K.T., Kostarelos K. (2011). Cytotoxic assessment of carbon nanotube interaction with cell cultures. Methods Mol. Biol..

[59] Goszczyński T.M., Fink K., Kowalski K., Leśnikowski Z.J., Boratyński J. (2017). Interactions of Boron Clusters and their Derivatives with Serum Albumin. Sci. Rep..

[60] Fuentes I., Pujols J., Viñas C., Ventura S., Teixidor F. (2019). Dual Binding Mode of Metallacarborane Produces a Robust Shield on Proteins. Chem. Eur. J..

[61] Moreno-Arriola E., Cárdenas-Rodríguez N., Coballase-Urrutia E., Pedraza-Chaverri J., Carmona-Aparicio L., Ortega-Cuellar D. (2014). Caenorhabditis elegans: A useful model for studying metabolic disorders in which oxidative stress is a contributing factor. Oxid. Med. Cell. Longev..

[62] Moreno-Arriola E., Cárdenas-Rodríguez N., Coballase-Urrutia E., Pedraza-Chaverri J., Carmona-Aparicio L., Ortega-Cuellar D. (2015). *C. elegans* as a tool for in vivo nanoparticle assessment. Adv. Colloid Interface Sci..

[63] Muñoz-Juan A., Assié A., Esteve-Codina A., Gut M., Benseny-Cases N., Samuel B.D., Dalfó E., Laromaine A. (2024). Caenorhabditis elegans endorse bacterial nanocellulose fibers as functional dietary Fiber reducing lipid markers. Carbohyd. Polym..

[64] Rojas S., Hidalgo T., Luo Z., Ávila D., Laromaine A., Horcajada P. (2022). Pushing the Limits on the Intestinal Crossing of Metal–Organic Frameworks: An Ex Vivo and In Vivo Detailed Study. ACS Nano.

[65] Revell S.H., Ishihara T., Sasaki M.S. (1983). Relationship between chromosome damage and cell death. Radiation-Induced Chromosome Damage in Man.

[66] Nomoto T., Inoue Y., Yao Y., Suzuki M., Kanamori K., Takemoto H., Matsui M., Tomoda K., Nishiyama N. (2020). Poly(vinyl alcohol) boosting therapeutic potential of p-boronophenylalanine in neutron capture therapy by modulating metabolism. Sci. Adv..

[67] Juárez-Pérez E.J., Granier M., Viñas C., Mutin P.H., Núñez R. (2012). Grafting of Metallacarboranes onto Self-Assembled Monolayers Deposited on Silicon Wafers. Chem. Asian J..

[68] Sramkova M., Kozics K., Masanova V., Uhnakova I., Razga F., Nemethova V., Mazancova P., Kapka-Skrzypczak L., Kruszewski M., Novotova M. (2019). Kidney nanotoxicity studied in human renal proximal tubule epithelial cell line TH1. Mutat. Res. Gen. Tox. En..

[69] González-Moragas L., Yu S.M., Carenza E., Laromaine A., Roig A. (2015). Protective Effects of Bovine Serum Albumin on Superparamagnetic Iron Oxide Nanoparticles Evaluated in the Nematode Caenorhabditis elegans. ACS Biomater. Sci. Eng..

[70] Brenner S. (1974). The genetics of Caenorhabditis elegans. Genetics.

