# Supplementary material for: Enhancing Boron Neutron Capture Therapy (BNCT) with Materials Based on COSAN-Functionalized Nanoparticles
Source: Pharmaceuticals (Basel). 2025 Mar 26;18(4):466. doi: 10.3390/ph18040466 (PMC12030506; doi:10.3390/ph18040466)
Supplement: Supplementary file 1 [file pharmaceuticals-18-00466-s001.zip › pharmaceuticals-3527177-supplementary.pdf]

Supporting Information

Enhancing Boron Neutron Capture Therapy (BNCT) with Materials Based on COSAN-Functionalized Nanoparticles

Albert Ferrer-Ugalde,[1] Amanda Muñoz-Juan,[1 † ‡] Anna Laromaine,[1] Paula Curotto,[2] Susana Nievas, [3] María Alejandra Dagrosa,[4] Marcos Couto*[5] and Rosario Núñez*[1]

1 Institut de Ciència de Materials de Barcelona (ICMAB-CSIC), Campus U.A.B., 08193 Bellaterra, Barcelona, Spain; a.ferrer80@hotmail.com (A.F.-U.); amunoz@mbg.au.dk (A.M.-J.); alaromaine@icmab.es (A.L.)

2 Department of Research and Production Reactors, National Atomic Energy Commission (CNEA), Presbitero Juan González y Aragón, 15, Ezeiza B1802AYA, Argentina; curotto@cae.cnea.gov.ar

3 Department of Boron Neutron Capture Therapy, National Atomic Energy Commission, Buenos Aires C1429BNP, Argentina; susanaisabelnievas@gmail.com

4 Department of Radiobiology, National Atomic Energy Commission (CNEA), Buenos Aires C1429BNP, Argentina; alejandradagrosa@cnea.gob.ar

5 Grupo de Química Orgánica Medicinal, Instituto de Química Biológica, Facultad de Ciencias, Universidad de la República, Iguá 4225, Montevideo 11400, Uruguay

† Current address: Department of Molecular Biology and Genetics, Aarhus University, 8000 Aarhus, Denmark.

‡ *Current address: Danish Research Institute of Translational Neuroscience—DANDRITE, Nordic-EMBL Partnership for Molecular Medicine, Aarhus University, 8000 Aarhus, Denmark.*

Table of contents

Figure S1. FT-IR spectrum of **NP@-I-COSAN**..S3

Figure S2. TGA of **NP@I-COSAN** (continuous line) and the starting compounds **NPs** (dashed line) and **I-COSAN** (dotted line). TGA were performed under flowing air at a heating rate of 10 ºC·min^{-1}……………………………………………………………………………………..S4

Figure S3. TEM images and EDX analysis of **NP@I-COSAN**..S5

Figure S4: UV-vis spectra of **NP@-ICOSAN** in EtOH and H_2O………………………………S6

Table S1. Selected TGA data ……………………………………………………………S7

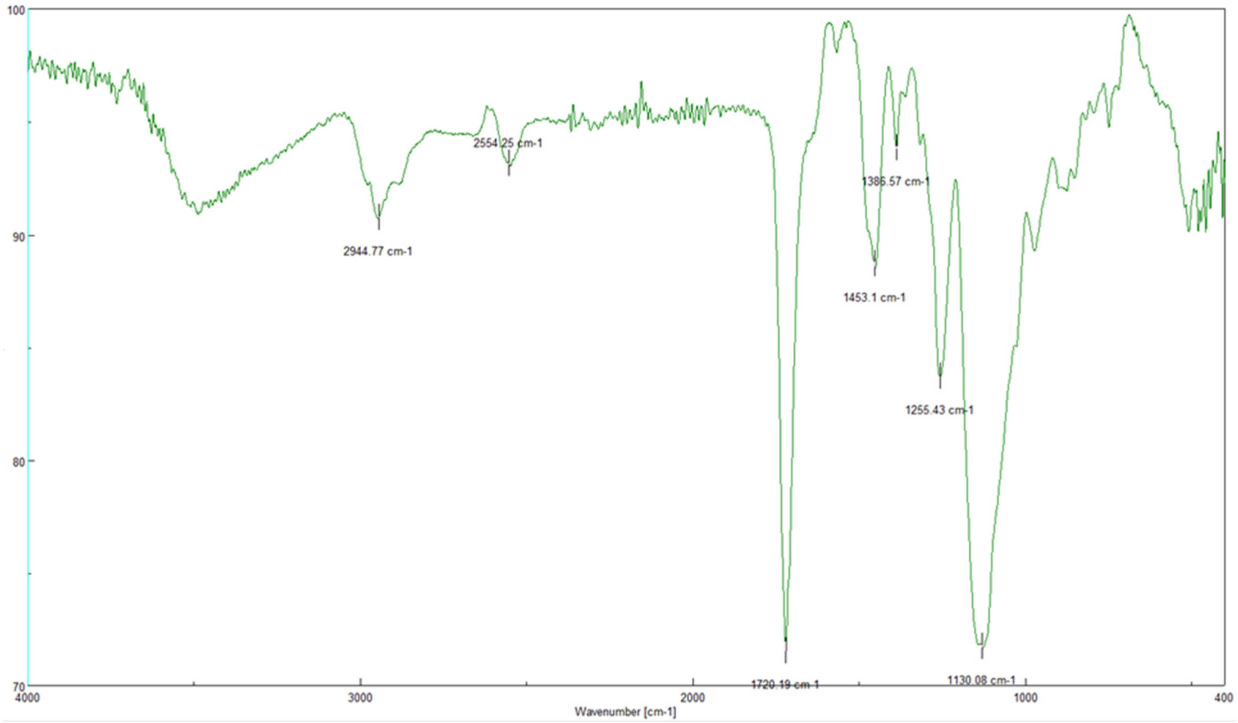

Figure S1. FT-IR spectrum of **NP@-I-COSAN**.

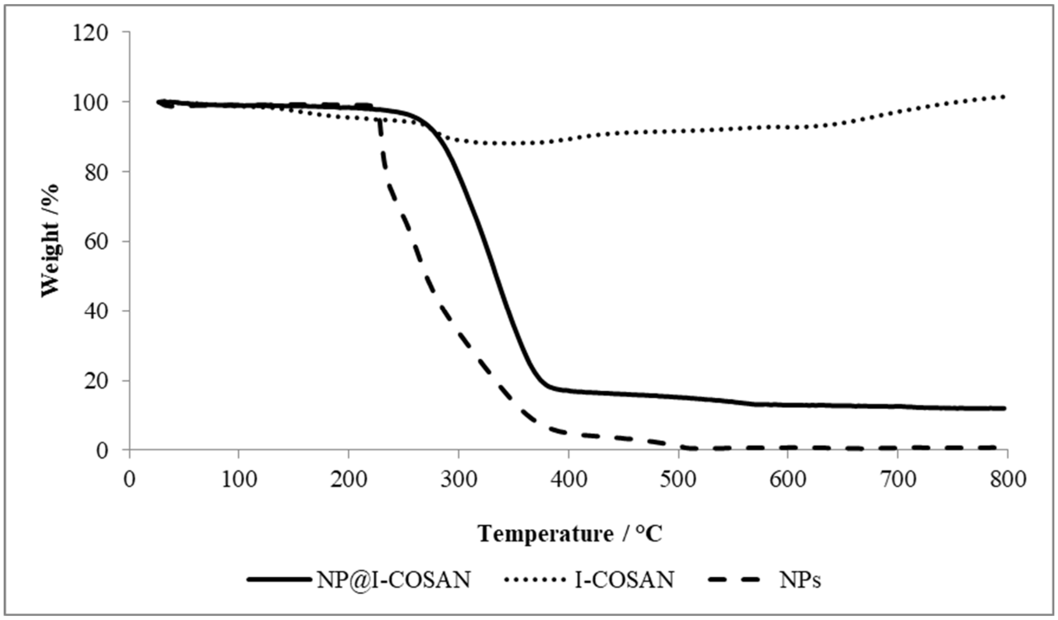

Figure S2. TGA of **NP@I-COSAN** (continuous line) and the starting compounds **NPs** (dashed line) and **I-COSAN** (dotted line). TGA were performed under flowing air at a heating rate of 10 ºC·min^{-1}.

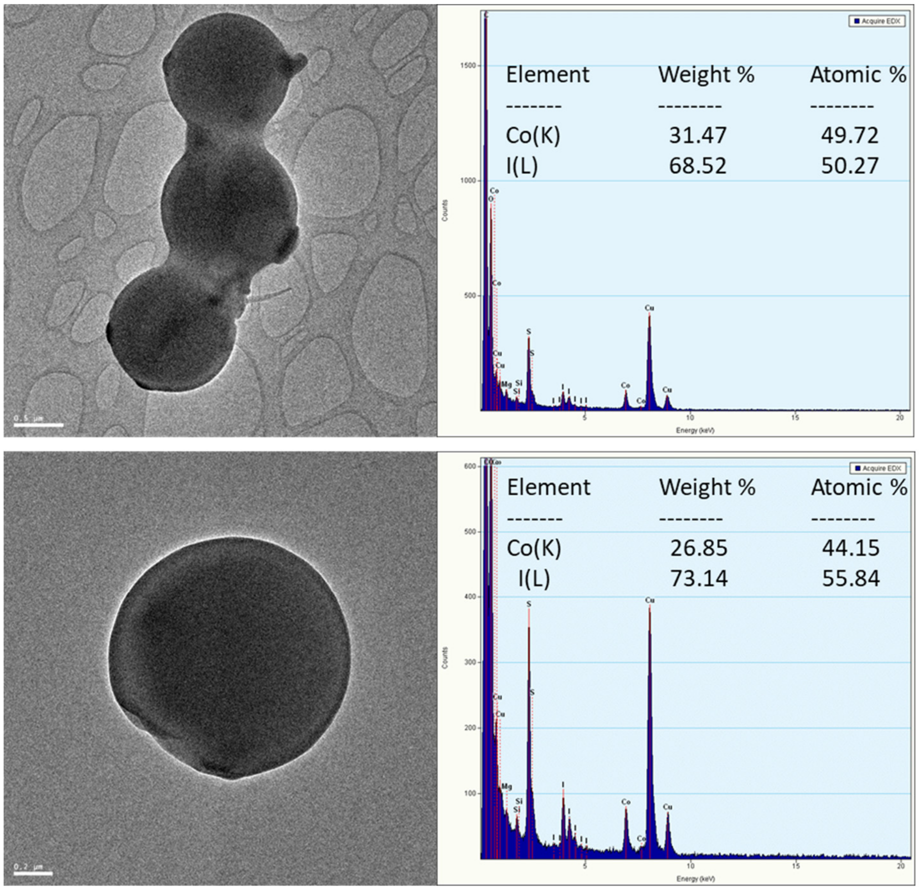

Figure S3. TEM images and EDX analysis of **NP@I-COSAN**.

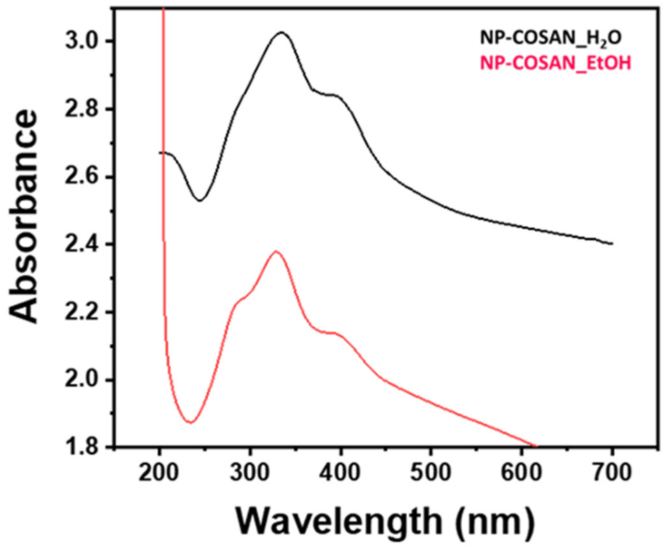

Figure S4: UV-vis spectra of **NP@-ICOSAN** in EtOH and H_2O

Table S1. Selected TGA data

**Compound**	**Residue at 800ºC (wt%)**	**Degree of functionalization (μmol·g^{-1})**
NPs	0.70	-
I-COSAN	101.55	-
NP@I-COSAN	14.29	253
